# Supplementary material for: P-TRAP: a Panicle Trait Phenotyping tool
Source: BMC Plant Biol. 2013 Aug 29;13:122. doi: 10.1186/1471-2229-13-122 (PMC3848748; doi:10.1186/1471-2229-13-122)
Supplement: Additional file 7 — P-TRAP source code. The Netbeans project that contains the source code of the application. [file 1471-2229-13-122-S7.zip › javadoc.zip!/javadoc/org-biolag-particleprocessor/org/biolag/particleprocessor/ParticleProcessor.html]

ParticleProcessor (ParticleProcessor)


JavaScript is disabled on your browser.


- Package
- Class
- Tree
- Deprecated
- Index
- Help

*org.biolag.particleprocessor 1.0*

- Prev Class
- Next Class

- Frames
- No Frames

- All Classes

- Summary:
- Nested |
- Field |
- Constr |
- Method

- Detail:
- Field |
- Constr |
- Method


org.biolag.particleprocessor

## Class ParticleProcessor

- java.lang.Object
- - org.biolag.particleprocessor.ParticleProcessor

- ---

    

  ```
  public abstract class ParticleProcessor
  extends java.lang.Object
  ```

  Is a set of algorithm for detecting particles on grayscale and colored images.

  Author:
  :   faroq

- - ### Field Summary

    Fields

    | Modifier and Type | Field and Description |
    | --- | --- |
    | `static int` | `counter` |
  - ### Constructor Summary

    Constructors

    Constructor and Description

    `ParticleProcessor()`
  - ### Method Summary

    Methods

    | Modifier and Type | Method and Description |
    | --- | --- |
    | `static ParticlesModel` | `getModels(java.io.File imagefile, int meanWindowSize, int c, int minparticleSize, boolean isDarkBackground)` Return a `ParticlesModel` of the detected particle in the input image. |
    | `static ParticlesModel` | `getModelsColored(java.io.File imagefile, int mindisk, int maxdisk, org.bioalg.imageprocessor.HSBPixel objectPixel, org.bioalg.imageprocessor.HSBPixel backPixel)` Grains finding in colored images using math morphology approach |
    | `static ParticlesModel` | `getModelsMathMorphology(java.io.File imagefile, int meanwindow, int mindisk, int maxdisk, boolean isDarkBackground)` Grains finding using math morphology approach |
    | `static ParticlesModel` | `getModelsRGBImage(java.io.File imagefile, org.bioalg.imageprocessor.HSBPixel objectPixel, org.bioalg.imageprocessor.HSBPixel backPixel, int minparticleSize)` Detect the particle by using the color thresholding approach. |
    | `static Particle` | `getPartcle(org.bioalg.imageprocessor.AImage image, java.awt.Point s, java.awt.Point e)` |
    | `static ParticlesModel` | `getXModels(java.io.File imagefile)` This is a general particles finder function that only smooth and threshold locally the source image then detect the particles. |
    | `static int` | `ispariclesinArea(org.bioalg.imageprocessor.AImage image, java.awt.Point start, java.awt.Point end, java.util.ArrayList<Particle> particles)` |

    - ### Methods inherited from class java.lang.Object

      `clone, equals, finalize, getClass, hashCode, notify, notifyAll, toString, wait, wait, wait`

- - ### Field Detail


    - #### counter

      ```
      public static int counter
      ```
  - ### Constructor Detail


    - #### ParticleProcessor

      ```
      public ParticleProcessor()
      ```
  - ### Method Detail


    - #### getPartcle

      ```
      public static Particle getPartcle(org.bioalg.imageprocessor.AImage image,
                        java.awt.Point s,
                        java.awt.Point e)
      ```


    - #### ispariclesinArea

      ```
      public static int ispariclesinArea(org.bioalg.imageprocessor.AImage image,
                         java.awt.Point start,
                         java.awt.Point end,
                         java.util.ArrayList<Particle> particles)
      ```


    - #### getXModels

      ```
      public static ParticlesModel getXModels(java.io.File imagefile)
      ```

      This is a general particles finder function that only smooth and
      threshold locally the source image then detect the particles.

      Parameters:
      :   `imagefile` - the input image

      Returns:
      :   a `ParticlesModel`


    - #### getModels

      ```
      public static ParticlesModel getModels(java.io.File imagefile,
                             int meanWindowSize,
                             int c,
                             int minparticleSize,
                             boolean isDarkBackground)
      ```

      Return a `ParticlesModel` of the detected particle in the input image.

      Parameters:
      :   `imagefile` - the image file.
      :   `meanWindowSize` - the width of the mean thresholding method window.
      :   `c` - the decline constant
      :   `minparticleSize` - the minimum number of pixels to accept as particle.
      :   `isDarkBackground` - true if the image have dark background.


    - #### getModelsRGBImage

      ```
      public static ParticlesModel getModelsRGBImage(java.io.File imagefile,
                                     org.bioalg.imageprocessor.HSBPixel objectPixel,
                                     org.bioalg.imageprocessor.HSBPixel backPixel,
                                     int minparticleSize)
      ```

      Detect the particle by using the color thresholding approach.

      Parameters:
      :   `imagefile` - the input file
      :   `objectPixel` - the color (`HSBPixel`) of the object.
      :   `backPixel` - the color (`HSBPixel`) of the background.
      :   `minparticleSize` - the minimum number of pixels to accept as particle.

      Returns:
      :   a `ParticlesModel` object


    - #### getModelsMathMorphology

      ```
      public static ParticlesModel getModelsMathMorphology(java.io.File imagefile,
                                           int meanwindow,
                                           int mindisk,
                                           int maxdisk,
                                           boolean isDarkBackground)
      ```

      Grains finding using math morphology approach

      Parameters:
      :   `imagefile` - the image file.

      Returns:
      :   a `ParticlesModel`


    - #### getModelsColored

      ```
      public static ParticlesModel getModelsColored(java.io.File imagefile,
                                    int mindisk,
                                    int maxdisk,
                                    org.bioalg.imageprocessor.HSBPixel objectPixel,
                                    org.bioalg.imageprocessor.HSBPixel backPixel)
      ```

      Grains finding in colored images using math morphology approach

      Parameters:
      :   `imagefile` - the

      Returns:


- Package
- Class
- Tree
- Deprecated
- Index
- Help

*org.biolag.particleprocessor 1.0*

- Prev Class
- Next Class

- Frames
- No Frames

- All Classes

- Summary:
- Nested |
- Field |
- Constr |
- Method

- Detail:
- Field |
- Constr |
- Method
